# Classification of Daily Body Weight Gains in Beef Calves Using Decision Trees, Artificial Neural Networks, and Logistic Regression

**DOI:** 10.3390/ani13121956

**Published:** 2023-06-11

**Authors:** Wilhelm Grzesiak, Daniel Zaborski, Renata Pilarczyk, Jerzy Wójcik, Krzysztof Adamczyk

**Affiliations:** 1 Department of Ruminants Science, West Pomeranian University of Technology, Klemensa Janickiego 29, 71-270 Szczecin, Poland; 2 Department of Genetics, Animal Breeding and Ethology, University of Agriculture in Krakow, al. Mickiewicza 24/28, 30-059 Kraków, Poland; krzysztof.adamczyk@urk.edu.pl

**Keywords:** classification, body weight gains, beef calves, decision trees, artificial neural networks, logistic regression

## Abstract

**Simple Summary:**

In the management of beef cattle, it can be useful to divide individuals based on a specific trait value (below and above average). This in turn allows for focusing on a larger group of animals with the aim of improving, e.g., their growth rate or obtaining a more uniform group in terms of a given trait. Classifying calves into less (below average) and more (above average) efficient growth creates an opportunity for producers to direct their efforts towards the “worse” animals and improve their performance through adjustments in nutrition, animal grouping, or reorganization of work. In this study, models were developed based on data from a beef farm. They were used to classify beef calves into poorer and better growth groups. In order to obtain more input data, predictions were made for the third calf. Among the analyzed models, random forest was the most effective. The most significant factors influencing daily body weight gains were also identified and discussed in the present study. The results demonstrate that machine learning models can be useful for classifying calves based on their growth rates. However, it is necessary to maintain proper breeding documentation from which the predictors can be obtained.

**Abstract:**

The aim of the present study was to compare the predictive performance of decision trees, artificial neural networks, and logistic regression used for the classification of daily body weight gains in beef calves. A total of 680 pure-breed Simmental and 373 Limousin cows from the largest farm in the West Pomeranian Province, whose calves were fattened between 2014 and 2016, were included in the study. Pre-weaning daily body weight gains were divided into two categories: A—equal to or lower than the weighted mean for each breed and sex and B—higher than the mean. Models were developed separately for each breed. Sensitivity, specificity, accuracy, and area under the curve on a test set for the best model (random forest) were 0.83, 0.67, 0.76, and 0.82 and 0.68, 0.86, 0.78, and 0.81 for the Limousin and Simmental breeds, respectively. The most important predictors were daily weight gains of the dam when she was a calf, daily weight gains of the first calf, sex of the third calf, milk yield at first lactation, birth weight of the third calf, dam birth weight, dam hip height, and second calving season. The selected machine learning models can be used quite effectively for the classification of calves based on their daily weight gains.

## 1. Introduction

The assessment of fattening intensity provides essential information for adjusting feed quantity, monitoring animal health [1,2], improving genetic selection efficiency [3], and determining optimal slaughter time [4], as animals that have already reached slaughter weight can become a financial burden for a farmer [5]. For the producer, it is important to achieve optimal body weight at slaughter within a specified time. It is also significant to “balance” the herd structure or the group of animals in terms of factors such as body weight gains, despite individual variations. Focusing on a larger group of animals to improve a certain trait is sometimes more desirable than paying special attention to single individuals, which in turn is typical of breeding.

Classifying calves into less (below average) and more (above average) efficiently growing categories allows the producer to direct efforts towards “inferior” animals in order to improve performance through adjustments in nutrition, animal grouping, reorganization of work, etc. Regular, at least monthly, body weight control by weighing of animals is a fundamental activity that determines fattening progress (growth rate analysis) and its completion. It should, however, be noted that weighing is a stressful activity for animals (especially under grazing conditions). It reduces their welfare and generates additional financial costs and organizational challenges for a farmer. However, it is possible to use alternative methods of body weight estimation and data processing by the application of certain statistical models [6,7]. Advanced technologies, including machine learning methods, can reduce human errors, increase farmers’ profits, improve farm productivity and animal welfare, and aid in the development of more holistic, humane, and environmentally friendly practices [8].

Predicting the magnitude of a trait (defined class) and determining the influence of various factors and rules that affect this prediction becomes possible with the use of classical statistical methods, the so-called machine learning methods, and artificial intelligence [9]. However, determining in advance which method will provide the highest accuracy can be challenging [10].

Decision trees are classification or regression models formulated in a tree-like architecture. The dataset is progressively organized into smaller, homogeneous subsets, forming a connected tree graph [11]. Each internal node of the tree structure represents a pairwise comparison of a selected trait. Each branch depicts the outcome of that comparison [12]. Leaf nodes represent the final decision or prediction made after traversing the path from the root to the leaf, expressed as a classification rule [13,14,15].

Decision trees, belonging to machine learning methods, allow for predicting the level of different traits while controlling for the factors affecting it. The application of these techniques in various zootechnical analyses is increasing [16,17,18,19,20]. The obtained information allows for identifying individuals with potentially low values of a given trait. This in turn enables actions towards improving this condition or indicating animals for culling [16]. The choice of decision trees is based on many useful aspects of their applicability, such as the clear explanation of the obtained solutions and the intuitiveness of interpretation [21]. Moreover, unlike artificial neural networks, the construction of a decision tree model is easy to follow. The quality of trees is not only determined by their predictive performance, but also by the splitting rules illustrating useful relationships [22]. Artificial neural networks (ANN), on the other hand, are a combination of multiple processing units that create a specific topology and mimic complex biological functions to solve regression or classification problems [15].

In terms of the previous studies on the application of statistical methods to daily weight gain prediction, Cominotte et al. [6] predicted body weight and average daily body weight gains in beef cattle based on three-dimensional images using multiple linear regression, least absolute shrinkage and selection operator, partial least squares, and ANN. The ANN was superior for predicting average daily body weight gains for the weaning to stocker, weaning to the beginning of feedlot, weaning to the end of feedlot, stocker to the beginning of feedlot, and beginning to the end of feedlot periods. Benedeti et al. [23] estimated and validated regression equations to predict carcass weight, empty body weight gain, and retained energy of Zebu cattle based on the independent variables such as slaughter plant, sex, genotype, shrunk body weight, carcass gain, and equivalent empty body weight. The authors stated that the equations accurately and precisely estimated empty body weight gain of Zebu and Zebu-cross cattle in the independent validation dataset based on daily carcass gain. Zhao et al. [7] applied a commercial software (Cornell Net Carbohydrate and Protein System) for predicting daily weight gains in Chinese local beef breeds based on an ingested metabolizable energy allowance automatically calculated by the computer model. The generated predictions were fairly accurate as evidenced by low biases between predicted and observed values. Lee et al. [24] used different machine learning methods (linear regression, tree regression, adaptive boosting, and a deep neural network) for predicting average daily weight gains in pigs based on temperature, humidity, feed intake, and the current animal weight. The applied algorithms were capable of predicting the trait accurately even despite the heterogeneity of the growth characteristics of pigs. In addition, ANNs were superior to other models. Osorio et al. [25] predicted average daily gains in lambs based on the digestibility and composition of the diet using a regression model and obtained acceptable accuracy and precision. Finally, Aranda et al. [26] utilized a deterministic model for predicting daily weight gains in growing steers grazing tropical pastures. It included the effects of protein and energy intake from forages and supplements. However, its accuracy was quite limited.

The aim of this study was to classify daily body weight gains in beef calves (below or above the regional average based on data recorded on a farm) using decision trees (classification and regression trees (CART), chi-square automatic interaction detection (CHAID) trees, and random forests (RF)). The obtained results were compared with another artificial intelligence method, i.e., ANN, and a classical statistical method, i.e., logistic regression (LR). The second aim of the present study was to identify the variables that contributed most to the classification of daily body weight gains.

The article is organized as follows: Section 2 describes materials and methods. Section 3 contains results. Section 4 discusses the results and describes related works in the same field. Section 5 includes the final conclusions and possible avenues for future work.

## 2. Materials and Methods

### 2.1. Data

Data from documentation and interviews conducted at the largest farm in north-western Poland (West Pomeranian Voivodeship) were used for analysis. Performance records were initially obtained for 1053 cows of two breeds (680 Simmental and 373 Limousin cows), whose calves were fattened between 2014 and 2016. Two data subsets were created separately for each breed, following the suggestion by Deodhar and Ghosh [27]. Only dams with three consecutive parturitions were selected. Due to incomplete (e.g., large number of missing cases for the third gestation length) or erroneous data (e.g., too high daily weight gains, too long gestation period, too high age at first calving, etc.), some questionable information was omitted. The final number of complete records amounted to 840 (527 and 313 for the Simmental and Limousin breeds, respectively). A description of Limousin and Simmental cows included in the study is presented in Table 1.

Beef cattle were kept in a rotation grazing system with voluntary use of shelters and fed a total mixed ration (TMR). The silage consisting mainly of corn, grass, alfalfa, and crop plants supplemented with vitamin–mineral premixes was fed twice a day. The management and feeding were consistent for all animals during the analyzed period (adjusted for animal age and body weight), and no significant changes or deviations that could affect body weight gains were observed. A more detailed description of the management system is provided by Pilarczyk and Wójcik [28].

The data from farm records about dams and their offspring for three successive calvings were included in the models (Table 2).

A total of 21 predictor variables were used. The classification (predicted) variable was the average daily body weight gain of the third calf of the cow, measured until its weaning at approximately 210 days of age. The gains were calculated from birth to the weaning of each calf. The weighted average was determined for calves of the same breed and sex from the two previous periods of evaluating beef cattle productive value in the breeding region where the research was conducted. For the purpose of classification, daily body weight gains of calves from the third calving of the cow (third production period) were divided into two categories (classes):

- Class A—the gains equal to or lower than the established weighted average body weight gains. This class represented calves with “worse” daily body weight gains.
- Class B—the gains higher than the established weighted average body weight gains. This class represented calves with “better” daily body weight gains.

The dataset was balanced for the Simmental (Class A—229 calves (43.45%) and Class B—298 calves (56.55%)) and Limousin (Class A—175 calves (55.73%) and Class B—139 calves (44.27%)) breeds.

The average daily weight gains for calves of a given breed and sex from the two previous periods of evaluating beef cattle productive value (successive production years) were obtained from the “Evaluation of Beef Cattle Productive Value” published by the Polish Association of Beef Cattle Breeders and Producers (https://bydlo.com.pl/ocena-wartosci-uzytkowej-bydla-miesnego/, accessed on 24 January 2023). They are presented in Table 3.

### 2.2. Preparing Predictive Models

The full data subset for each breed was randomly divided into a training set and a test set for the development and verification of individual models (395 records in the training set and 132 in the test set for the Simmental breed; 234 records in the training set and 79 in the test set for the Limousin breed; Table 4).

The same training set was used to prepare the LR, ANN, and decision tree (CART, CHAID, and RF) models. Similarly, the same test set was used to verify the predictive performance of each model, i.e., their ability to classify (detect) daily body weight gains of calves above and below the average daily body weight gain determined for a given breed and sex. In the process of hyper-parameter tuning, the default values were used as the basis for each model. They were subsequently modified in order to check whether model performance improved. Model selection was based on a validation set or a 10-fold cross-validation. In the case of ANN, the so-called automatic network designer was used to find the best model among multilayer perceptrons (MLP) with one and two hidden layers, radial basis function (RBF), and linear networks. The total number of the analyzed networks was 200, whereas activation functions for the hidden and output neurons included linear, logistic, hyperbolic tangent, and exponential ones. The sum of squares and cross-entropy were used as error functions and weight reduction was performed from 0.001 to 0.01. For RF, the optimal number of component trees was determined on the validation set, whereas the 10-fold cross-validation was applied to find the best CART, CHAID, and LR models. The final parameters for individual models are presented in Table 5.

### 2.3. Predictive Performance

When selecting models for detection, the classification matrix (Table 6) was used to calculate sensitivity (*SEN*), specificity (*SPF*), and accuracy (i.e., overall error probability- *ACC*) according to the following equations:

$$SEN=\frac{TP}{TP+FN},\;SPF=\frac{TN}{FP+TN}\;,\;ACC=\frac{TP+TN}{TP+FP+FN+TN}\;$$

where:

*SEN*—sensitivity, which is the probability of correctly classifying Class A weight gains out of all weight gains in Class A;

*SPF*—specificity, which is the probability of correctly classifying Class B weight gains out of all weight gains in Class B;

*ACC*—overall error probability, which is the overall accuracy of prediction;

*TP*—the number of correctly classified weight gains below the average;

*FN*—the number of incorrectly classified weight gains below the average;

*FP*—the number of incorrectly classified weight gains above the average;

*TN*—the number of correctly classified weight gains above the average.

The importance of individual variables for decision trees and RF is presented as a percentage relative to the most important variable. Each stage of division and the contribution of each variable were analyzed. Variables were subsequently assigned ranks (the most important variable received the lowest rank, but the ranks were reversed on the chart for better readability, so that the bar for the most important variable is the highest). The importance of variables to the LR model was primarily analyzed based on their statistical significance, and the ranks were subsequently assigned as for the models above.

For ANN, predictor importance was determined using sensitivity analysis, taking into account the ratio and rank of each variable. The ratio should be interpreted as follows: if the value for a given variable is above one, removing that variable from the training set may result in decreased model quality, and vice versa: values below one indicate that the contribution of the variable is insignificant. The chart shows the five most important variables for each model.

### 2.4. Model Comparison

When comparing model quality, the above-mentioned indicators (*SEN*, *SPF*, *ACC*) were considered. Statistically significant differences between them were tested using the McNemar test with the Bonferroni correction. Receiver operating characteristic (ROC) curves were also generated for each model. They show the decision at the optimal cutoff point based on the values of the dependent variable dividing the set of cases into two groups (with gains below and above the average). The curve allows for the assessment of classification quality for the generated models and comparison among them. The points obtained from the calculations are plotted on a coordinate system and connected by a line to form the ROC curve. The area under the ROC curve (AUC) within the range of 0 to 1 informs about the quality of assigning cases to a specific group. The AUC was calculated according to the most widely recommended DeLong’s method [29] and indicates the discriminatory ability of the model. A good classifier is the one that correctly recognizes and groups cases into a specific class, being characterized by the highest AUC value close to unity.

Matthews correlation coefficient (*MCC*) was also calculated as a complement to the above criteria:

$$MCC=\frac{\left(TP\cdot TN\right)-\left(FN\cdot FP\right)}{\sqrt{\left(TP+FN\right)\left(TP+FP\right)\left(FN+TN\right)\left(FP+TN\right)}}$$

This correlation coefficient is based on true positives and false positives as well as true negatives and false negatives. It is a balanced measure even when individual classes have different sample sizes. *MCC* ranges from −1 to +1, where values close to −1 indicate perfect misclassification and those close to +1 show excellent classification. Values around 0 suggest random classification [30,31].

Statistical analyses were performed using Statistica software (v. 13.3, Tibco Inc., Tulsa, OK, USA 2018) and Statistica Neural Networks program (StatSoft Inc., Tulsa, OK, USA).

## 3. Results

The CART and CHAID models obtained in the present study are presented in Figure S1. Table S1 summarizes their quality indicators (*SEN*, *SPF*, *ACC*, and *MCC*) calculated on the training set for the Limousin and Simmental breeds. Table S2 presents the number of cases in classification matrices obtained for the training set. 

For the Limousin breed, RF had the highest sensitivity (0.83) and CHAID had the lowest one (0.55). The sensitivity of other models ranged from 0.72 for ANN to 0.78 for CART. It was statistically significantly different only from the lowest sensitivity of the CHAID model (Table 7). The specificity ranged from 0.59 for LR to 0.77 for CHAID, although the differences were not statistically significant. The accuracy ranged from 0.64 for CHAID to 0.76 for RF.

For the Simmental breed, ANN had the highest sensitivity (0.77), while LR had the lowest one (0.66). The sensitivity of other models was similar (0.73 for CHAID, 0.70 for CART, and 0.68 for RF). These values did not differ significantly. The RF model had the highest specificity (0.86), followed by CART (0.80). These values were statistically significantly different from those for other models (0.67 for LR, 0.62 for CHAID and ANN).

The RF model also had the highest overall accuracy (0.78), while the LR and CHAID models had the lowest one (0.67). These differences were statistically significant. The *ACC* values for ANN and CART were 0.69 and 0.76, respectively (Table 7). The respective classification matrices are shown in Table S3.

The *MCC* values for the Limousin breed ranged from 0.33 for CHAID to 0.52 for RF, but these differences were not statistically significant. For the Simmental breed, the *MCC* values ranged between 0.33 for LR and 0.55 for RF. Significant differences (*p* < 0.05) were observed between the *MCC* values for RF and LR, as well as RF and CHAID (Table 7).

**Figure 1:**
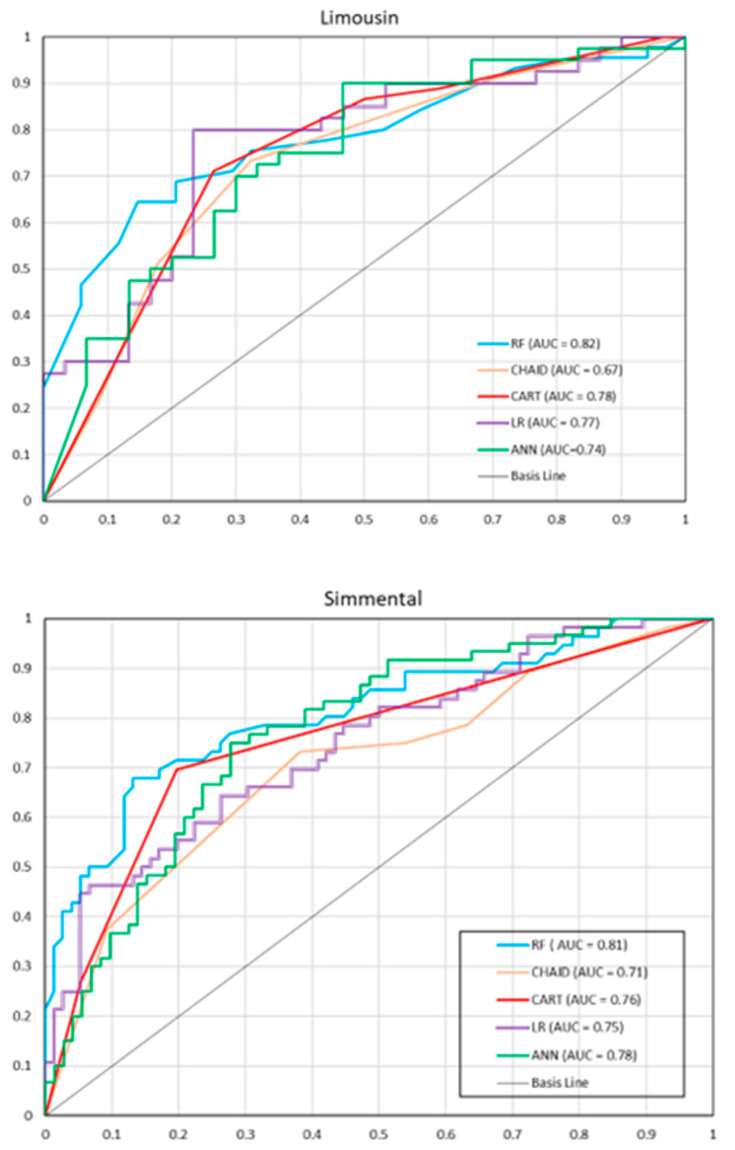
Receiver operating characteristic (ROC) curves for individual models on the test set (the Limousine and Simmental breed). RF—random forest, CHAID—chi-square automatic interaction detector, CART—classifications and regression trees, LR—logistic regression, ANN—artificial neural network.

ROC curves with AUC values for individual models are presented in Figure 1. For the Limousin breed, RF had the largest AUC (0.82), indicating that it was the best model based on this criterion. CHAID had the smallest AUC (0.67). For other models, the AUC values ranged from 0.74 to 0.78. Similar AUC values were observed for the Simmental breed. RF had the highest AUC (0.81), while CHAID had the lowest one (0.71). The AUC values for other models ranged from 0.75 to 0.78.

### Predictor Importance

**Figure 2:**
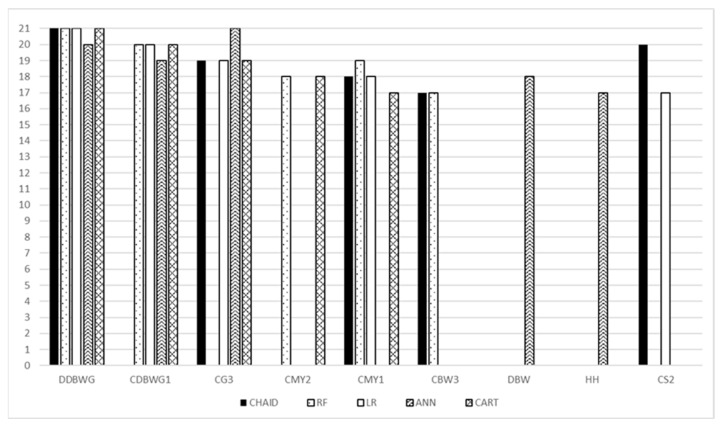
Comparison of the top five most important variables for individual models. DDBWG—daily body weight gains of the dam when she was a calf; CDBWG1—daily body weight gains for the first calf; CG3—sex of the third calf; CMY2—milk yield from calving to weaning at second lactation; CMY1—milk yield from calving to weaning at first lactation; CBW3—birth weight of the third calf; DBW—dam birth weight; HH—hip height; CS2—second calving season; RF—random forest, CHAID—chi-square automatic interaction detector, CART—classifications and regression trees, LR—logistic regression, ANN—artificial neural network.

For each model, the most important predictor was daily body weight gains of the dam when she was a calf (DDBWG). Daily body weight gains for the first calf (CDBWG1) were included among the top five most important predictive variables for all models, except CHAID. Sex of the third calf (CG3) was important for all models, except RF. Additionally, milk yield from calving to weaning at first lactation (CMY1) was important for all models, except ANN. The remaining variables (milk yield from calving to weaning at second lactation (CMY2), birth weight of the third calf (CBW3), dam birth weight (DBW), hip height (HH), and second calving season (CS2)) were important for some models (Figure 2). The full set of predictor variables with their ranks is presented in the Supplementary Material Table S4.

## 4. Discussion

The novelty of our approach consisted in the use of routinely collected on-farm data for daily body weight gain prediction. The second advantage was the prediction of daily body weight gains in beef cattle (and not body weight itself). There are few studies about daily weight gain prediction in cattle, especially using machine learning methods. Finally, the classification of daily weight gains was applied in our study, which enabled animal grouping.

### 4.1. Model Quality and Predictive Performance

The analysis of two separate data sets, divided by breed, resulted from the heterogeneity of classification accuracy in data segments, as indicated by Magidson [32] and Ratner [33]. Due to an insufficient sample size, the data set was not further divided according to sex. The performance indicators obtained on the test set for individual models were generally lower than those on the training set. Exceptions were sensitivity, accuracy, and *MCC* for CART used for Simmental cattle, which slightly increased compared to those on the training set. The sensitivity was also somewhat higher for CHAID. For Limousin cattle, higher *MCC* values were observed for LR and CART (Table 7).

In the case of daily body weight gains of both Limousin and Simmental calves, RF had the highest sensitivity (0.92 and 0.73, respectively) (Table S1). However, the sensitivity on the test set for the RF model used for the Simmental breed decreased and was lower (although not significantly) than that for other models (Table 7). RF had high specificity, which was statistically significantly different from that for LR, CHAID, and ANN.

In the present study, the sensitivity for CHAID used for the Limousin breed was significantly lower (0.55) compared to other models, while that for the Simmental breed was higher (0.73), which may confirm the suggestions made by Antipov and Pokryshevskaya [34] that the CHAID trees, due to the use of multi-way splits, require relatively large sample sizes.

The qualitative parameters of the models analyzed in the present study indicate, in light of the available literature, a fairly good quality. From a practical point of view, sensitivity, which is the ability of the model to identify lower body weight gains, plays a more important role in this case. Early identification of animals with such gains allows farmers to apply various preemptive measures to eliminate unfavorable consequences and improve individuals with lower body weight gains.

Accuracy on the test set for RF, CHAID, CART, ANN, and LR was similar and ranged from 0.68 to 0.75. Generally, it can be stated that the quality of LR, decision trees, and ANN obtained in the present study did not substantially differ from that reported by other authors. Additional analysis of the models included ROC curves and AUC. Depending on the model, this area ranged from 0.67 to 0.82. The higher the AUC (compared to the area under the line y = x corresponding to AUC = 0.5), the better the classification quality [35]. It should be noted that the obtained AUC value for the RF was the highest, although in some cases, the parameters for RF were slightly lower than those for other models (e.g., sensitivity for the Simmental breed was the highest for ANN). This suggests certain inconsistency and contradiction in recommending the appropriate model. Some researchers suggest that AUC should be considered as a secondary criterion for model selection. This would imply that for the Simmental breed, ANN would be better than RF, but on the other hand, the differences in sensitivity were not statistically significant.

Another performance indicator was *MCC*, whose values were generally average, ranging from 0.33 to 0.55 depending on the breed. The value of this criterion favored RF. These values were similar to those obtained by others. As indicated by Luque et al. [36], when classification errors are of great importance, *MCC* is the best measure of model performance.

CHAID has a visual and easy-to-interpret output; however, due to the use of multiway splits, it requires large sample sizes for effective prediction [37]. CART, on the other hand, is a binary decision tree, which is constructed by recursively splitting a node into two child nodes, starting from the root node, which contains the entire training sample. CART aims to maximize homogeneity within a node [38]. Finally, LR is a well-known and easy-to-interpret method, which provides an advantage over other approaches (e.g., neural networks). It can yield good and reliable results in comparative studies [39] and may outperform more sophisticated methods [40]. On the other hand, ANNs, similar to CHAID, generally require larger datasets.

### 4.2. Predictive Variables in the Models

There are many factors affecting average daily body weight gains. These can be genetic (breed) and environmental (habitat, nutrition, management, disease, weather) ones [2] or their interaction. The weather factors include ambient temperature, wind speed, solar radiation, and dew point [9]. Among the variables used in the present study, the daily body weight gains of the dam when she was a calf (DDBWG) had the greatest importance. The higher the body weight gain of the young dam in the first days after birth, the higher the probability that she will complete her first and subsequent lactations. Increasing the body weight gains of heifer calves from birth to weaning can increase the milk production of reared animals in the first lactation [41,42]. This information supports the need for paying more attention to heifer calves. Monitoring their daily body weight gains results in their proper growth and development, potentially higher milk production in the future, and thus better offspring rearing. This variable was indicated by all models and seems to be crucial for the future development of a cow’s offspring.

Equally important were the variables associated with the daily body weight gains for the first calf and the dam milk yield for the first and second calf. Higher dam milk yield potentially leads to increased daily body weight gains of her calf [43]. This indicates that some cows have a predisposition for rearing calves with high body weight gains. Information about the body weight gains for the first calf and the dam milk yield affects the body weight gains for the third calf of that dam, providing a valuable insight into the future potential of beef calves of these breeds already in the first production cycle. The sex of the third calf of the dam was also an important indicator of body weight gains. Generally, the body weight gains of bull calves are higher than those of heifer calves, which is confirmed by other studies as well [28]. The birth weight of the third calf was also noted to have an effect on body weight gains, albeit only for the CHAID and RF models. The birth weight of the calf may affect the body weight gains, as it is related to the milk yield of the dam, which in turn influences the body weight gains of the calves [44]. The effect of some variables was observed for ANN, i.e., dam birth weight (DBW) and hip height (HH). Han et al. [45] found that among similarly managed heifers, heavier animals at first calving produced slightly more milk in the first lactation than lighter ones, and more milk results in higher body weight gains of the calves. Hip height (HH) may be correlated with reproductive traits and body weight [46], which in turn may affect milk yield and calf body weight gains. The influence of calving season on body weight gains may also be observed. Calves born in the winter achieve higher daily body weight gains than young cattle born in the spring [47]. Casasús et al. [48] found that cows that calved in the fall had significantly better calf rearing results than those calving in the spring. This was due to better pasture utilization for milk production and increased milk yield during the feeding period. Cows that calved in the summer gave birth to calves with lower body weight, which was later reflected in their growth [49]. Not all variables included in the analysis were necessary to construct a particular model. The above-described variables were found to have large contribution to determining calf body weight gains, however, it cannot be excluded that the order of variables’ importance would be different for a different dataset. The variable daily body weight gains of the dam when she was a calf (DDBWG), which played a crucial role in each of the presented models, needs to be highlighted.

An interesting option of decision trees is their ability to split sample and gradually group animals with specific trait values, which is presented in the Supplementary Material. It is additional information about the influence of individual traits on decision tree models. Besides model quality, knowledge of successive splits at different stages of tree construction can also be important (e.g., daily body weight gains of the dam when she was a calf (DDBWG) for the CHAID model includes two crucial body weight gain values—981 and 904 g/d—and milk yield from calving to weaning at first lactation for RF—2158 kg).

Finally, limitations of the present study should be briefly mentioned. The sample size used for model training was relatively small. Additionally, not all predictors included in the machine learning models are routinely recorded on all farms, especially zoometric measurements. Moreover, the prediction pertains to the daily weight gains of the third calf of the cow while maintaining continuity (i.e., provided that the data for the first and second calf of the same dam are available). In future research, a larger sample size, additional predictors, and other types of predictive models should be used.

### 4.3. Related Work

Considering other related studies, Bowen et al. [50], analyzing early detection of respiratory diseases in calves using moving average and RF models, obtained lower sensitivity (0.35–0.43), high specificity (0.95–0.97), and average accuracy (0.64–0.65), which was comparable to the values in the present study. After applying a combined moving average/RF model, the authors achieved increased sensitivity (0.54) and accuracy (0.75), while specificity remained mostly unchanged. Becker et al. [51], predicting heat stress in dairy cattle using the RF, LR, and naive Bayes classifier (NBC) models, obtained, in general, higher sensitivity (0.83–0.93, depending on the group), specificity (0.78–0.89), and accuracy (0.81–0.89). Uckardes et al. [52], using CHAID for fertility analysis of Japanese quails, reported a sensitivity of 0.82, whereas Kramer et al. [53] and Kamphuis et al. [54], detecting mastitis in cows, obtained a sensitivity of 0.75 and specificity of approximately 0.92 to 0.99. Piwczyński et al. [55] developed a decision tree model for dystocia detection in dairy cows with a much lower sensitivity (0.61) than that in the present study. Pietersma et al. [56], using CART for lactation milk yield analysis, recorded a sensitivity of only 0.45, whereas Ortiz-Pelaez and Pfeiffer [57], studying disease prevalence in Welsh cattle, obtained a very high sensitivity (0.91) and a relatively low specificity (0.36), which indicates quite a good model for the detection of ill individuals.

Similar accuracy for the RF model was obtained by Shahinfar et al. [58] in predicting fertilization results in cows (0.72 for primipara and 0.74 for multipara), as well as by Montgomery et al. [59] and Yang et al. [60] in mastitis detection (0.75 and 0.67–0.77, respectively, depending on the type of model used). Basarab et al. [61] recorded the *ACC* values of 0.68–0.93 in dystocia detection in heifers. Very high *ACC* (0.96) was also reported by Pastell and Kujala [62] when identifying lameness in cattle, while much lower accuracy (0.32) was observed by Abell et al. [63] in detecting lameness in sows.

In terms of AUC, Grzesiak et al. [16] obtained lower values of this parameter (0.64 for CART and 0.74 for ANN) in predicting body weight gains of calves. On the other hand, Tambuyzer et al. [64] reported the same AUC value as that for the CHAID model in the present study when predicting the survival of infected pigs. Similar AUC values were also recorded by Pastell and Kujala [62] in classifying healthy and lame cows. Heirbaut et al. [65] obtained AUC values ranging from 0.69 to 0.81 for different RF models constructed to predict metabolic clusters of cows during early lactation based on somatic cell count in milk. They were quite similar to those in the present study, which confirms the comparable classification abilities of the models.

Finally, Bowen et al. [50] reported significantly lower *MCC* values (0.25–0.29) for a dataset with a high degree of diversity (imbalanced). After combining two models, MCC values increased to 0.36. Higher *MCC* (0.6–0.8) was found by Becker et al. [51], who applied ANN, among others, for heat stress classification in cattle; however, the results were significantly worse than those for other methods.

## 5. Conclusions

The present study showed that the applied predictive models based on farm-available data had the ability to fairly accurately classify daily body weight gains in the third production cycle. They showed high sensitivity in predicting lower gains, moderate specificity (ability of the model to correctly identify higher body weight gains), balanced accuracy, and moderate *MCC* values. The RF model performed the best in terms of various quality indicators, with most of them being higher than those for other models, whereas the CHAID model exhibited the worst predictive performance. LR and ANN also performed well. However, decision trees and ANN have an advantage over LR, because they do not require assumptions of their applicability. Among the 21 variables, several of the most important ones can be utilized, namely daily body weight gains of the dam when she was a calf, daily body weight gains for the first calf, sex of the third calf (from the third pregnancy), milk yield from calving to weaning at first lactation, milk yield from calving to weaning at second lactation, birth weight of the third calf, dam birth weight, hip height, and second calving season, although not for all models, each of which requires an individual approach. It should be mentioned that random forest can be used for the preliminary processing of on-farm collected data to assess the chances of dams’ offspring for low or high body gains according to the adopted criterion.

In the future, predictors that turned out to be less important in the present study could be excluded from the models, which would facilitate their practical application on beef farms. On the other hand, the current predictive performance could be increased by including additional predictors (not utilized in the present study), such as animal welfare parameters (e.g., temperature–humidity index), the occurrence of (chronic) diseases, the proportion of animals that died during the fattening period, economic factors related to the producer’s decisions about fattening management (e.g., the ratio of livestock prices to feed prices), and those associated with animal feeding. These new variables would, however, require additional measurements to be taken. It is also recommended to develop ensemble models (based on majority voting), which would integrate decision trees, artificial neural networks, logistic regression, etc., in order to further improve predictive performance. Taking into account prediction results for beef calves obtained in the present study, similar models could also be used for classifying daily weight gains in other beef breeds for different production regions. Such a classification system may be extended to other livestock species (e.g., pigs) and combined with dressing percentage prediction. Finally, the developed models could be implemented in a computer program.

## Figures and Tables

**Table 1:** Selected morphometric parameters and performance indicators for the examined dams grouped by breed.

Breed	*n*	HH (cm)	WH (cm)	CHG (cm)	DBW (kg)	WW (kg)	WA (d)	DDBWG (g)
Limousin	313	136.2	134.9	196.5	31.7	260	226	1010
Simmental	527	139.6	135.9	197.1	34.7	260	227	993

HH—hip height, WH—withers height, CHG—chest girth, DBW—dam birth weight, WW—weaning weight, WA—weaning age, DDBWG—dam daily body weight gains.

**Table 2:** Predictors for daily body weight gain classification.

Predictor	Abbreviation	Unit	Type
Dam birth weight	DBW	kg	M
Daily body weight gains of the dam when she was a calf	DDBWG	kg	M
Dam’s hip height	HH	cm	M
Dam’s withers height	WH	cm	M
Dam’s chest girth	CHG	cm	M
First calving season *	CS1	-	C
First gestation length	GL1	d	M
Birth weight of the first calf	CBW1	kg	M
Sex of the first calf (from the first pregnancy)	CG1	-	C
Milk yield from calving to weaning at first lactation	CMY1	kg	M
Daily body weight gains of the first calf	CDBWG1	g/d	M
Dam’s age at first calving	AFC	mo	M
Second calving season *	CS2	-	C
Second gestation length	GL2	d	M
Birth weight of the second calf	CBW2	kg	M
Sex of the second calf (from the second pregnancy)	CG2	-	C
Milk yield from calving to weaning at second lactation	CMY2	kg	M
Daily body weight gains of the second calf	CDBWG2	g/d	M
Third calving season *	CS3	-	C
Birth weight of the third calf	CBW3	kg	M
Sex of the third calf (from the third pregnancy)	CG3	-	C
Class of body weight gains for the third calf **	CBWC3	g/d	C

* Two calving seasons were distinguished: spring–summer (April to September) and autumn–winter (October to March); ** predicted variable; M—measurable variable; C—categorical variable.

**Table 3:** Weighted average daily body weight gains (g) during the study period for Limousin and Simmental heifer and bull calves in the West Pomeranian Province.

Sex	2014	2015	Average
Limousin			
Heifer calves	976	1024	999.68
Bull calves	1039	1117	1078.24
Simmental			
Heifer calves	1100	1130	1114.38
Bull calves	1288	1201	1241.86

**Table 4:** Number of calves for both breeds.

Breed	Training Set	Test Set	Total
Limousin	234	79	313
Simmental	395	132	527

**Table 5:** Model parameters.

Model	Validation Method	Misclassification Costs	Prior Probabilities	Remaining Parameters
CART	10-fold cross-validation	Equal	Estimated from the training set	Goodness-of-fit criterion: Gini index, stopping rule: misclassification error-based pruning; stopping parameters: minimum node size = 52 (31 for the Limousin breed), maximum number of nodes = 1000
CHAID	10-fold cross-validation	Equal	-	Stopping parameters: minimum node size = 94, maximum number of nodes = 1000, splitting and merging probability = 0.05, Bonferroni correction
RF	Validation set (25%)	Equal	Estimated from the training set	Number of randomly selected predictors = 5, maximum number of trees = 250 (150 for the Limousin breed), proportion of random subsamples = 0.50, initial value of random number generator = 1; stopping parameters: minimum node size = 7 (5 for the Limousin breed), maximum number of levels = 10, minimum child node size = 5, maximum number of nodes = 100; advanced training stop criterion: a 5% decrease in an error rate for 10 cycles
ANN	Validation set (15%—Limousin, 25%—Simmental)	Equal	-	Network type: MLP with two hidden layers (seven and two neurons, respectively) for the Simmental breed, MLP with one hidden layer (sixteen neurons) for the Limousin breed; training algorithm: BFGS; activation functions: logistic (for the hidden neurons), linear (for the output neuron); error function: sum of squares
LR	10-fold cross-validation	Equal	-	Qualitative variable encoding: sigma limits (quasi-experimental); model building method: all effects included (maximum number of iterations = 100); statistical tests: Wald, Pearson’s χ^2^; significance levels for predictors = 0.05

RF—random forest, CHAID—chi-square automatic interaction detector, CART—classifications and regression trees, LR—logistic regression, ANN—artificial neural network, MLP—multilayer perceptron, BFGS—Broyden–Fletcher–Goldfarb–Shanno.

**Table 6:** Classification matrix.

Predicted	Observed	
	Class A (Lower Gains)	Class B (Higher Gains)
Class A (lower gains)	*TP*	*FP*
Class B (higher gains)	*FN*	*TN*

**Table 7:** Model performance indicators and Matthews correlation coefficients (*MCC*) on the test set for the individual models.

Model	Limousin				Simmental			
	*SEN*	*SPF*	*ACC*	*MCC*	*SEN*	*SPF*	*ACC*	*MCC*
LR	0.76 ^B^	0.59	0.68	0.35	0.66	0.67 ^Bb^	0.67 ^b^	0.33 ^b^
CART	0.78 ^B^	0.70	0.74	0.48	0.70	0.80 ^ACa^	0.76	0.50
CHAID	0.55 ^Aa^	0.77	0.64	0.33	0.73	0.62 ^BC^	0.67 ^b^	0.35
RF	0.83 ^B^	0.67	0.76	0.52	0.68	0.86 ^A^	0.78 ^a^	0.55 ^a^
ANN	0.73 ^b^	0.67	0.70	0.39	0.77	0.62 ^BC^	0.69	0.38 ^b^

LR—logistic regression, CART—classification and regression trees, CHAID—chi-square automatic interaction detector, RF—random forest, ANN—artificial neural networks, *SEN*—sensitivity, *SPF*—specificity, *ACC*—accuracy, *MCC*—Matthews correlation coefficient; values with different superscript letters differ at *p* ≤ 0.05 (small letters) and *p* ≤ 0.01 (capital letters).

## Data Availability

The data presented in this study are available on request from the corresponding author. The data are not publicly available due to confidentiality.

## Supplementary Materials

The following supporting information can be downloaded at https://www.mdpi.com/article/10.3390/ani13121956/s1, Table S1: Quality indicators and Matthews correlation coefficients for the individual models based on the training set, Table S2: Classification matrices for the individual models based on the training set, Table S3: Classification matrices for the individual models based on the test set, Table S4: The sums of ranks for the predictors included in the individual models (inverted ranks); Figure S1: CART model for the Limousin breed (part 1), CHAID model for the Limousin breed (part2), CART model for the Simmental breed (part 3), CHAID model for the Simmental breed (part 4).
