# Supplementary material for: Classification of Daily Body Weight Gains in Beef Calves Using Decision Trees, Artificial Neural Networks, and Logistic Regression
Source: Animals (Basel). 2023 Jun 11;13(12):1956. doi: 10.3390/ani13121956 (PMC10295584; doi:10.3390/ani13121956)
Supplement: Supplementary file 1 [file animals-13-01956-s001.zip › animals-2378193-supplementary.pdf]

animals

MDPI

Supplementary Material

Table S1. Quality indicators and Matthews correlation coefficients for the individual models based on the training set (or combined validation and training set).

Model	Limousin				Simmental			
	SEN	SPF	ACC	MCC	SEN	SPF	ACC	MCC
**LR**	0.82	0.65	0.75	0.28	0.73^{B}	0.78^{B}	0.76^{b}	0.51
**CART**	0.81	0.74	0.78	0.47	0.59^{A}	0.83^{B}	0.73^{b}	0.44
**CHAID**	0.71^{b}	0.77	0.74	0.38	0.72^{B}	0.71^{Aa}	0.71^{b}	0.43^{b}
**RF**	0.92^{a}	0.65	0.80	0.55	0.73^{B}	0.89^{B}	0.82^{a}	0.63^{a}
**ANN**	0.84	0.79	0.82	0.52	0.72^{B}	0.77^{b}	0.74^{b}	0.48

LR – logistic regression, CART – classification and regression trees, CHAID – chi-square automatic interaction detector, RF – random forest, ANN – artificial neural networks, SEN – sensitivity, SPF – specificity, AC – accuracy, MCC – Matthews correlation coefficient

Table S2. Classification matrices for the individual models based on the training set (or combined validation and training set).

	Limousin									
	LR		CART		CHAID		RF		ANN	
	A	B	A	B	A	B	A	B	A	B
A	93	37	106	24	88	42	113	17	108	33
B	46	59	37	68	31	74	35	70	22	72
	Simmental									
	LR		CART		CHAID		RF		ANN	
	A	B	A	B	A	B	A	B	A	B
A	126	47	102	71	124	49	127	46	124	49
B	48	174	37	185	64	158	25	197	52	170

LR – logistic regression, CART – classification and regression trees, CHAID – chi-square automatic interaction detector, RF – random forest, ANN – artificial neural networks, A – category A, B – category B

Table S3. Classification matrices for the individual models based on the test set.

	Limousin									
	LR		CART		CHAID		RF		ANN	
	A	B	A	B	A	B	A	B	A	B
A	34	14	35	10	20	10	35	12	30	13
B	11	20	10	24	16	33	7	25	11	25
	Simmental									
	LR		CART		CHAID		RF		ANN	
	A	B	A	B	A	B	A	B	A	B
A	37	25	39	15	41	29	38	11	43	29
B	19	51	17	61	15	47	18	65	13	47

LR – logistic regression, CART – classification and regression trees, CHAID – chi-square automatic interaction detector, RF – random forest, ANN – artificial neural networks, A – category A, B – category B

Table S4. The sums of ranks for the predictors included in the individual models (inverted ranks).

	CHAID	RF	LR	ANN	CART	Total sum
**DDBWG**	1	1	1	2	1	**104**
**CDBWG1**	6	2	2	3	2	**95**
**CMY1**	4	3	4	6	5	**88**
**CG3**	3	16	3	1	3	**84**
**CMY2**	14	4	6	10	4	**72**
**CBW3**	5	5	8	16	8	**68**
**DBW**	13	8	9	4	12	**64**
**AFC**	10	7	20	8	6	**59**
**CBW2**	16	12	11	9	10	**52**
**CS2**	2	18	5	13	20	**52**
**WH**	9	9	7	17	18	**50**
**CDBWG2**	12	6	21	11	11	**49**
**HH**	17	14	10	5	16	**48**
**CBW1**	7	11	15	18	15	**44**
**GL1**	15	15	18	7	14	**41**
**CG1**	8	20	13	20	9	**40**
**CG**	19	10	14	15	13	**39**
**CS3**	11	21	16	12	21	**29**
**CS1**	20	19	12	14	17	**28**
**CG2**	21	17	19	21	7	**25**
**GL2**	18	13	17	19	19	**24**

LR – logistic regression, CART – classification and regression trees, CHAID – chi-square automatic interaction detector, RF – random forest, ANN – artificial neural networks

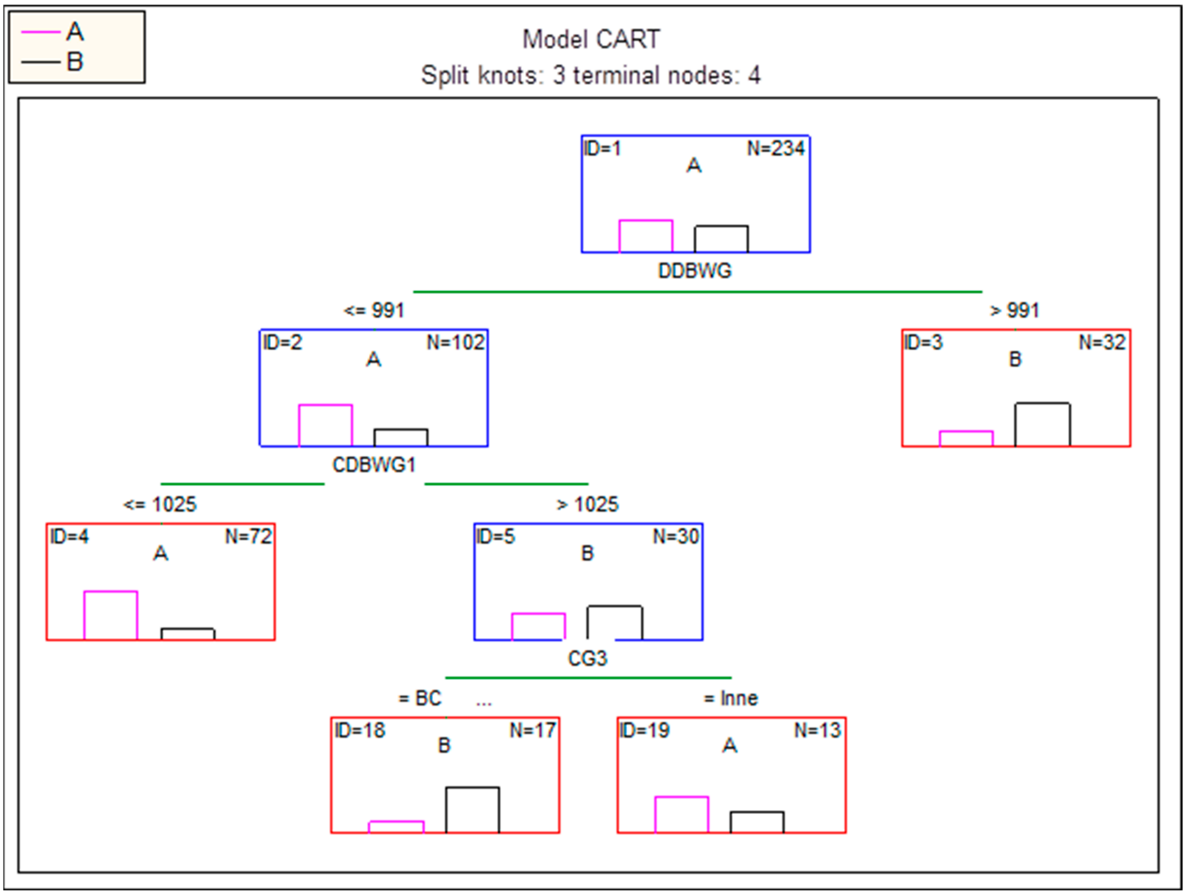

Figure S1. – part 1. CART model for the Limousin breed.

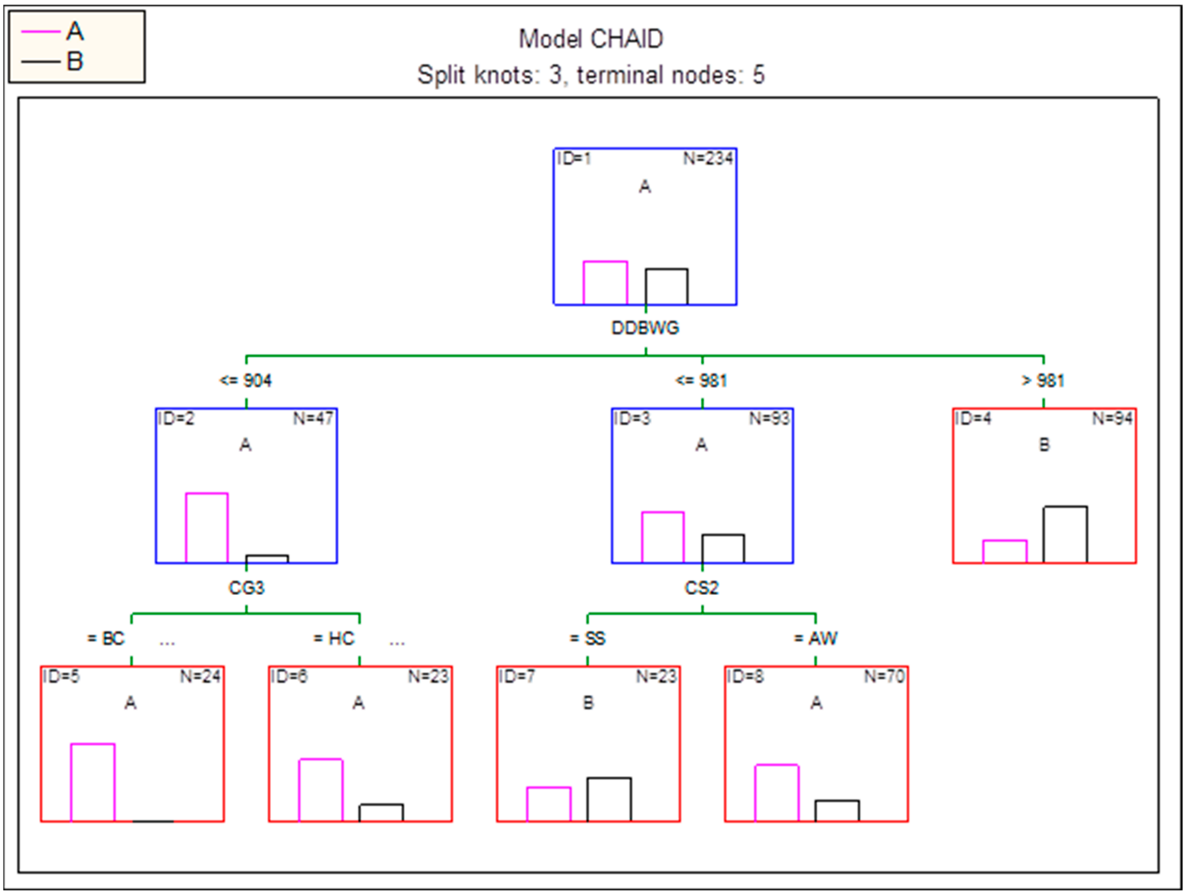

Figure S1. – part 2. CHAID model for the Limousin breed.

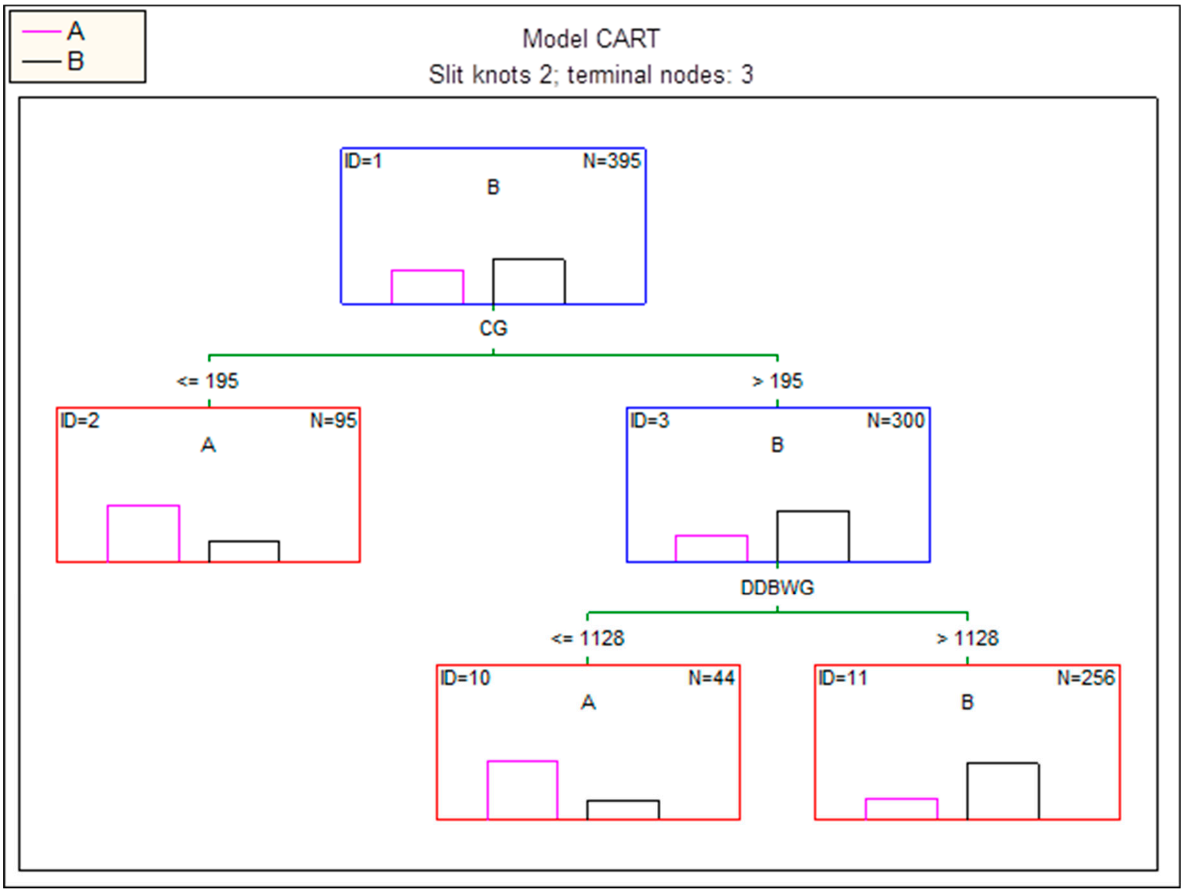

Figure S1. – part 3. CART model for the Simmental breed.

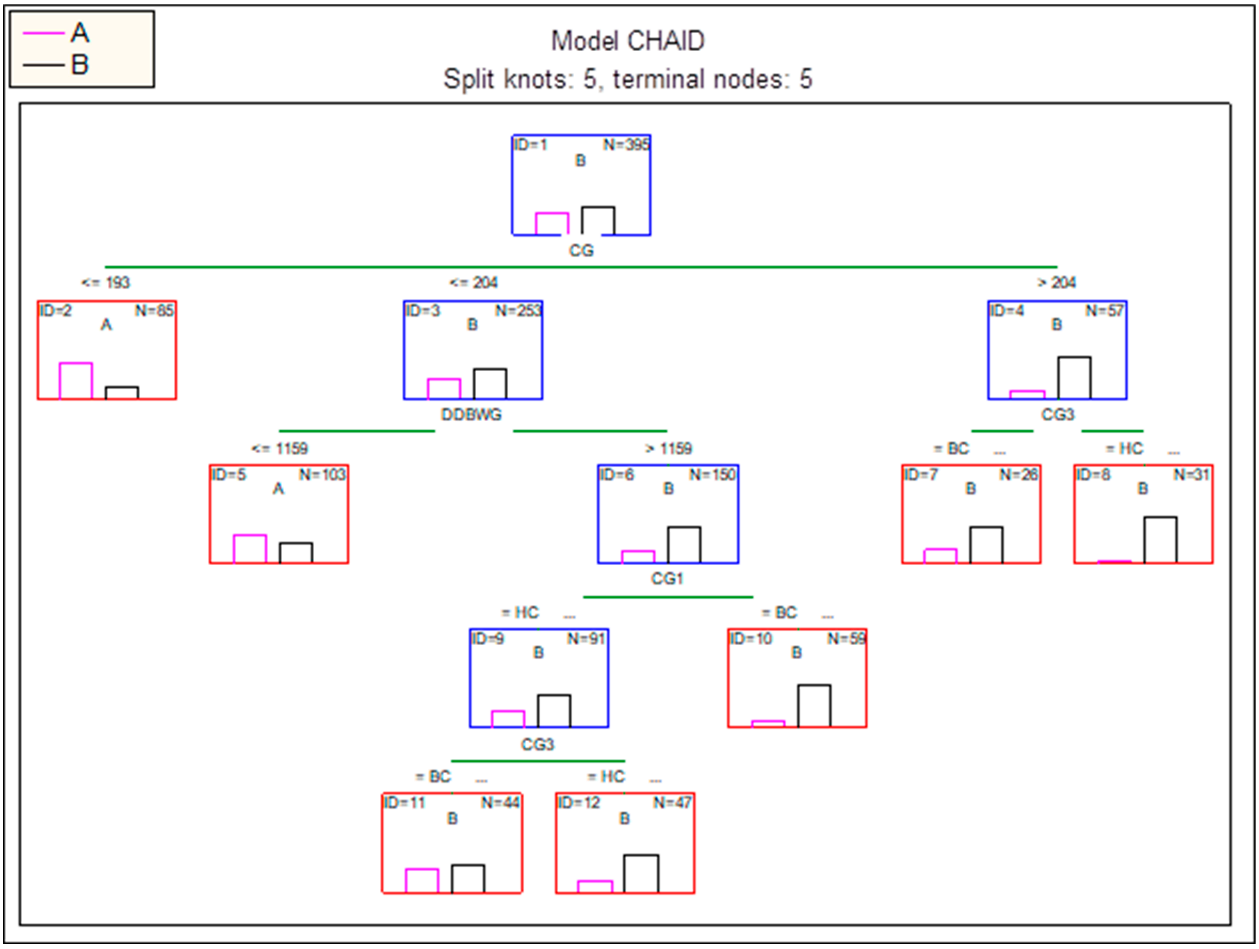

Figure S1. – part 4. CHAID model for the Simmental breed.
